# Genetic analysis of Verticillium wilt resistance in a backcross inbred line population and a meta-analysis of quantitative trait loci for disease resistance in cotton

**DOI:** 10.1186/s12864-015-1682-2

**Published:** 2015-08-05

**Authors:** Jinfa Zhang, Jiwen Yu, Wenfeng Pei, Xingli Li, Joseph Said, Mingzhou Song, Soum Sanogo

**Affiliations:** Department of Plant and Environmental Sciences, New Mexico State University, Las Cruces, NM 88003 USA; State Key Laboratory of Cotton Biology, Institute of Cotton Research of China, Chinese Academy of Agricultural Science, Anyang, Henan 455000 China; Department of Computer Science, New Mexico State University, Las Cruces, NM 88003 USA; Department of Entomology, Plant Pathology and Weed Science, New Mexico State University, Las Cruces, NM 88003 USA

**Keywords:** Cotton, Verticillium wilt, Fusarium wilt, Root-knot nematodes, Reniform nematodes, Resistance, Quantitative trait loci, Meta-analysis

## Abstract

**Background:**

Verticillium wilt (VW) and Fusarium wilt (FW), caused by the soil-borne fungi *Verticillium dahliae* and *Fusarium oxysporum* f. sp. *vasinfectum*, respectively, are two most destructive diseases in cotton production worldwide. Root-knot nematodes (*Meloidogyne incognita*, RKN) and reniform nematodes (*Rotylenchulus reniformis*, RN) cause the highest yield loss in the U.S. Planting disease resistant cultivars is the most cost effective control method. Numerous studies have reported mapping of quantitative trait loci (QTLs) for disease resistance in cotton; however, very few reliable QTLs were identified for use in genomic research and breeding.

**Results:**

This study first performed a 4-year replicated test of a backcross inbred line (BIL) population for VW resistance, and 10 resistance QTLs were mapped based on a 2895 cM linkage map with 392 SSR markers. The 10 VW QTLs were then placed to a consensus linkage map with other 182 VW QTLs, 75 RKN QTLs, 27 FW QTLs, and 7 RN QTLs reported from 32 publications. A meta-analysis of QTLs identified 28 QTL clusters including 13, 8 and 3 QTL hotspots for resistance to VW, RKN and FW, respectively. The number of QTLs and QTL clusters on chromosomes especially in the A-subgenome was significantly correlated with the number of nucleotide-binding site (NBS) genes, and the distribution of QTLs between homeologous A- and D- subgenome chromosomes was also significantly correlated.

**Conclusions:**

Ten VW resistance QTL identified in a 4-year replicated study have added useful information to the understanding of the genetic basis of VW resistance in cotton. Twenty-eight disease resistance QTL clusters and 24 hotspots identified from a total of 306 QTLs and linked SSR markers provide important information for marker-assisted selection and high resolution mapping of resistance QTLs and genes. The non-overlapping of most resistance QTL hotspots for different diseases indicates that their resistances are controlled by different genes.

**Electronic supplementary material:**

The online version of this article (doi:10.1186/s12864-015-1682-2) contains supplementary material, which is available to authorized users.

## Background

Upland cotton (*Gossypium hirsutum L.*, 2n = 4x = 52), as a tetraploid cotton, produces 97 % of lint fibers for the textile industry in the world, while extra-long staple (ELS) cotton (*G. barbadense L.*, 2n = 4x = 52), also known as Sea-Island, American Pima, or Egyptian cotton accounts for about 3 % of the world cotton. However, various diseases cause substantial yield losses in cotton [1–3]. Verticillium wilt (VW), caused by the soil-borne fungus *Verticillium dahliae* Kleb., and Fusarium wilt (FW), caused by the soil-borne fungus *Fusarium oxysporum* f. sp. *vasinfectum* (Atk.) Synd. & Hans, are two most destructive diseases in cotton production in the world. VW and FW can significantly reduce cotton yield and fiber quality due to leaf chlorosis, necrosis or wilting, leaf and boll abscission and plant death [4, 5]. Root-knot nematodes [*Meloidogyne incognita* (Kofoid & White), RKN] and reniform nematodes (*Rotylenchulus reniformis* Linford & Oliveira, RN) cause the highest yield loss (4.3 and 2.5 %, respectively) in the U.S. [3]. Planting disease resistant cultivars is the most effective and economical control method.

Many *G. barbadense* genotypes are known to carry high levels of resistance to VW [6–8], but its resistance has not been successfully transferred into commercial Upland cotton due to hybrid breakdown except for introgressed breeding lines [7, 9]. There have been many studies indicating that the VW resistance in *G. barbadense* is controlled by a dominant or partially dominant gene in interspecific crosses between *G. barbadense* and *G. hirsutum*. In several recent studies, more than 100 VW resistance quantitative trait loci (QTLs) in the interspecific Upland × Pima populations and also intra- Upland populations have been detected on almost all of the 26 tetraploid cotton chromosomes (c1 through c26) except for c2, c6, c10, c12, and c18, and VW resistance QTLs were more frequently detected on c5, c7, c8, c11, c16, c17, c19, c21, c23, c24, and c26 (for a review, see [4]).

A number of qualitative genetic studies have identified five major resistance genes against FW in Upland (in the U.S. and China) and Pima (in the U.S., Egypt and Israel) including *Fw1*, *Fw2*, *Fw*^*R*^ (c17), *FOV1* (c16) and *FOV4* (c14). There are also numerous quantitative genetic studies using early segregating populations confirming the predominant presence of additive gene effects with low heritabilities on FW resistance. Several recent mapping studies have collectively detected approximately 40 QTLs on all the 26 tetraploid cotton chromosomes except for c1, c4, c5, c10, c13, c20 and c24 (for a review, see [10]).

In Upland cotton, high RKN resistance was achieved in Auburn 623RKN through crossing between moderately resistant Clevewilt and Wild Mexico Jack Jones, which was then transferred to other breeding lines [11]. The resistance was later determined to be controlled by two genes through a classic genetic analysis [12], and the two genes (*Mi1* or *qMi-C11* and *Mi2* or *qMi-C14*) were mapped to chromosomes c11 from Clevewilt and c14 from Wild Mexico Jack Jones through a collective research effort [13, 14]. In an interspecific recombinant inbred line population of two susceptible parents of Upland and Pima cotton, major QTLs (on c3, c4, c11, c14, c17 and c23) and 19 putative QTLs for RKN responses were reported [15].

For RN, the high level of resistance (essentially immunity) in *G. longicalyx* was transferred to Upland cotton [16], and the resistance is conferred by a single dominant gene *Ren*^*lon*^ located on chromosome c11 [17]. Moderate resistance was also found in several *G. barbadense* accessions including GB 713 [18]. Three resistance QTLs (2 on c21 and 1 on c18) were identified in GB 713 in a cross with Acala Nem-X using SSR markers [19], and one of the two QTLs on c21 was later identified as a major QTL through SNP mapping [20]. In a cross between a tri-species hybrid *G. arboreum* × (*G. hirsutum* × *G. aridum*) and Upland MD51ne, a major dominant resistance gene *Ren*^*ari*^ also on c21 presumably from *G. aridum* was identified using SSR markers [21].

The consistency and utility of most resistance QTLs in breeding and genomic research identified for the above four major diseases remain uncertain. Because most QTL studies used early segregating populations such as F_2_, BC_1_F_1_ and F_2:3_, disease resistance could not be repeatedly evaluated for the same genotypes. However, QTLs as reported from different studies provide a good opportunity to perform a meta-analysis of resistance QTLs for identification of consistent resistance QTLs for the same disease (hotspots) and common QTLs for different diseases (clusters) across different studies. QTL clusters for resistance to different diseases and resistance QTL hotspots for the same disease will be very useful for breeders and geneticists to choose chromosome regions for marker-assisted selection and high resolution mapping of disease resistance QTLs or genes.

It is currently known that plant disease resistance is often conferred by disease resistance (R) genes including predominantly nucleotide-binding site (NBS)- encoding genes [22, 23]. R genes have evolved and clustered on the plant genome through various mechanisms such as tandem and segmental gene duplications, recombination, unequal crossing-over, point mutations, and diversifying selection. Recent genome sequencing studies have identified 391 and 280 NBS-encoding genes in *G. raimondii* and *G. arboreum*, respectively [24–26]. However, the relationship between the distribution of the NBS genes and resistance QTLs or genes is unclear in cotton.

The objectives of this study were to perform a QTL analysis of VW resistance from multiple years of replicated tests on a backcross inbred line (BIL) population of an interspecific Upland × Pima cross and a meta-analysis of QTLs and genes resistant to VW, FW, RKN and RN identified and reported previously. Relationships between the resistance QTLs and NBS genes on cotton chromosomes were also analyzed.

## Results

### QTL mapping for VW resistance in the BIL population

**Fig. 1:**
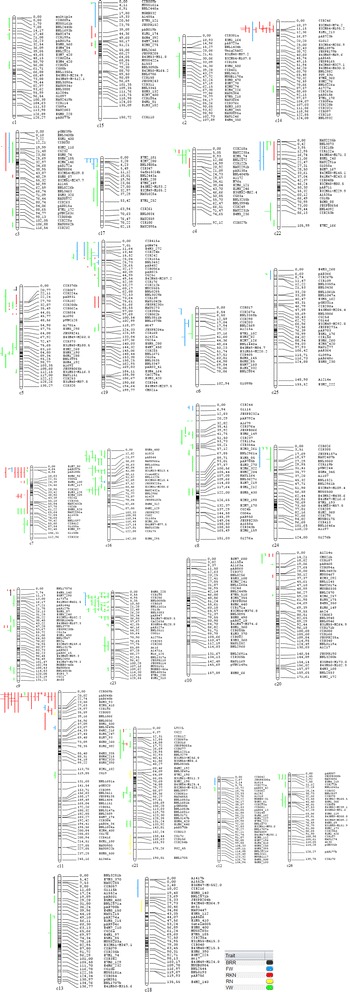
A meta-analysis of quantitative trait loci (QTLs) for resistance to Verticillium wilt (VW), Fusarium wilt (FW), root-knot nematodes (RKN), reniform nematodes (RN), and black root rot (*BRR*). When older markers with multiple positions along a chromosome were used as the sole means to position a QTL and the markers did not appear in the cotton marker database those QTL were not able to be placed on the (“Guazuncho2” (*G. hirsutum*) x “VH8-4602” (G*. barbadense*)) map. For this reason some QTL identified from previous studies were not included in this meta-analysis and do not appear on the combined QTL linkage map

In the BIL population of 146 lines tested in four years, we detected a total of 10 QTLs for VW resistance based on a linkage map with 392 polymorphic SSR loci spanning a total genetic distance of 2,895 cM as established by Yu et al. [27]. The QTLs were distributed on 8 chromosomes (Table 1, Additional file 1). These QTLs included two detected in 2006 and 2007 each, five in 2008 and one in 2009. Two VW-QTLs were located on chromosome c2 within a 25-cM region, while another two QTLs were on c4 in the same region with the same marker interval. Other six QTLs were located on c9, c12, c13, c21, c22, and c23 with one QTL each. The VW resistance QTLs were further mapped onto a consensus map (Fig. 1), which shows that VW resistance QTLs on these eight chromosomes were also reported by others previously.

**Table 1:** QTL for Verticillium wilt (VW) resistance detected in a backcross inbred line (BIL) population of 146 lines derived from a cross of (SG 747 × Giza 75) × SG 747 BC_2_F_4_

Year	QTL name	Position (cM)	Marker interval	LOD	Add.	PVE (%)	Direction
2006	qVWI-06-c2-1	2	NAU3775a-BNL0663	3.32	6.23	9.90	SG 747
2008	qVWI-08-c2-1	27	NAU3684-BNL3971	2.93	−8.59	32.38	Giza 75
2008	qVWR-08-c4-1	7	BNL3089-NAU3469	3.82	−0.19	35.43	Giza 75
2008	qVWI-08-c4-1	7	BNL3089-NAU3469	4.19	−7.95	41.98	Giza 75
2007	qVWI-07-c9-1	59	NAU3358-NAU5494	4.22	9.54	25.04	SG 747
2007	qVWR-06-c12-1	16	CIR272-NAU3401b	3.25	0.21	10.15	SG 747
2009	qVWI-09-c13-1	74	NAU2730-NAU5110	2.83	−11.56	25.07	Giza 75
2006	qVWI06-c21-1	106	NAU3341a-NAU3895	4.42	5.72	17.17	SG 747
2008	qVWI-08-c22-1	87	BNL0206-NAU3392	2.86	−7.53	40.39	Giza 75
2008	qVWR-09-c23-1	80	NAU5508-NAU3967	2.99	−0.15	14.18	Giza 75

*Add.* additive effect, *PVE* phenotypic variance explained, *VWI* VW incidence, *VWR* VW rating, c2, c4, c9, c12, c13, c21, c22 and c23, chromosomes

As shown in Table 1, the LOD scores for these QTLs ranged from 2.83 to 4.42, significant based on permutation tests, and phenotypic variation explained (PVE) by each QTL ranged from 9.9 to 42.0 %. Six QTLs that were from the resistance parent Giza 75 decreased Verticillium wilt incidents or severity ratings with higher PVE (14.2–42.0 %), while other four QTLs from the susceptible parent SG 747 increased Verticillium wilt incidents or severity ratings with lower PVE (9.9–25.0 %).

### Clusters of disease resistance QTLs

A total of 306 disease resistance QTLs have been reported including 10 VW resistance QTLs identified in this study (Table 2), with 193 VW resistance QTLs accounting for 63 % of the disease QTLs identified in past studies, followed by RKN (75) and FW (27). Chromosomes c23 carried the most resistance QTLs (36), followed by c7 (25 QTLs), c11 (23 QTLs), c21 (22 QTLs), c5 (21 QTLs), c16 (21 QTLs), and c19 (21 QTLs). Chromosomes c4, c8, c9, and c14 each carried 10–17 QTLs; c3, c6, c13, c15, c17, c20, c22, and c26 each carried 6–10 QTLs; c10 carried no resistance QTLs; and the remaining chromosomes (c1, c2, c12, c18, c24 and c25) carried the least number of QTLs (2–5).

**Table 2:** Numbers of quantitative trait loci (QTLs) resistant to Verticillium wilt (VW), Fusarium wilt (FW), root-knot nematodes (RKN), reniform nematodes (RN), bacterial blight (BB) and black root rot (BRR) that were identified in this study and reported in other studies

Chromosome	VW	FW	RKN	RN	BB	BRR	Total
c1	4	0	1	0	0	0	5
c2	5	0	0	0	0	0	5
c3	5	1	3	0	0	0	9
c4	8	0	2	0	0	0	10
c5	12	0	7	0	1	1	21
c6	5	3	0	0	0	0	8
c7	11	1	13	0	0	0	25
c8	12	2	0	0	0	0	14
c9	12	0	4	0	0	1	17
c10	0	0	0	0	0	0	0
c11	2	2	18	1	0	0	23
c12	2	1	0	0	0	0	3
c13	5	0	0	0	0	1	6
c14	3	1	9	0	1	0	14
c15	3	1	4	0	0	0	8
c16	20	1	0	0	0	0	21
c17	3	4	0	0	0	0	7
c18	0	1	0	1	0	0	2
c19	11	4	6	0	0	0	21
c20	4	0	3	0	0	0	7
c21	16	0	2	5	0	0	22
c22	6	2	1	0	0	0	9
c23	33	1	2	0	0	0	36
c24	3	1	0	0	0	0	4
c25	1	1	0	0	0	0	2
c26	7	0	0	0	0	0	7
Total	193	27	75	7	2	3	306

For the distribution of resistance QTLs on homeologous chromosomes, four pairs carried more QTLs including three pairs with similar numbers (c5 with 21 QTLs vs. c19 with 21 QTLs; c7 with 25 QTLs vs. c16 with 21 QTLs; and c11 with 23 vs. c21 with 23). However, c23 carried twice QTLs than its homeologous counterpart c9 (36 vs. 17). Four pairs carried less but with similar numbers of QTLs (c1 with 5 vs. c15 with 8; c3 with 9 vs. c17 with 7; c4 with 10 vs. c22 with 9; and c13 with 6 vs. c18 with 2). Among other five pairs carrying a few QTLs, three A-subgenome chromosomes carried less (c2 with 5 vs. c14 with 14; c10 with none vs. c20 with 7; and c12 with 3 vs. c26 with 7); and two other A-subgenome chromosomes carried more (c6 with 8 vs. c25 with 2; and c8 with 14 vs. c24 with 4). However, the number of QTLs carried between homeologous chromosomes was significantly and positively correlated (*r* = 0.682; 0.01 < *P* < 0.05). Overall, the A-subgenome carried 146 QTLs, which was slightly fewer than the D-subgenome (160). However, the difference was not statistically significant (*χ*^2^ = 0.64; χ^2^_0.05_ = 3.84 at df = 1).

Based on the selection criterion that 4 QTLs within a 25-cM region constitute a cluster, 28 QTL clusters (12 on the A subgenome- c1 to c13 and 16 on the D subgeome- c14 to c26) on 18 chromosomes (7 on the A subgenome and 11 on the D subgenome) were identified (Table 3), and they contained 210 resistance QTLs (accounting for ~70 % of the total resistance QTLs identified). The difference in distribution of clusters on the A- and D- subgenomes was not significant (*χ*^2^ = 0.57; χ^2^_0.05_ = 3.84 at df = 1). The first region (at 0–25 cM) on the chromosomes of the cotton genome contained most QTL clusters (16), followed by the second region at 25–50 cM with 8 clusters; and the third region at 60–85 cM and the fourth region at 75–100 cM only carried 2 clusters each. Across the cotton genome, the difference in distribution of clusters in the four regions was highly significant (*χ*^2^ = 18.86; χ^2^_0.05_ = 7.82 and χ^2^_0.01_ = 16.27 at df = 3). There were no clusters on c1, c2, c3, c10, c12, c13, c18, and c25. Chromosomes c7 and c16 each carried 3 clusters, while c5, c8, c11, c19, c21, and c23 each carried 2 clusters. However, some of the clusters did not contain any QTL hotspot (at least 4 resistance QTLs for the same disease) including c8 (2 VW and 2 FW QTLs at 0–20 cM), c9 (3 VW and 2 RKN QTLs at 35–55 cM), c20 (3 VW and 2 RKN QTLs at 0–20 cM), c22 (2 VW, 2 FW and 1 RKN QTL at 0–25 cM) and c24 (3 VW and 1 FW QTL at 0–20 cM). Some of the clusters may contain QTL hotspots after more QTL mapping results are reported in the future. In the following sections, clusters containing resistance QTL hotspots for each disease will be analyzed in more details.

**Table 3:** Resistance QTL clusters and hotspots identified for Verticillium wilt (VW), Fusarium wilt (FW), rootknot nematodes (RKN) and reniform nematodes (RN)

Chr	Total	No. clusters	No. QTLs	Type hotspot	Region	Linked markers
c1	5	0				
c2	5	0				
c3	9	0				
c4	10	1	5 VW, 2 RKN	VW	0–10 cM	CIR 210A, NAU2235a, BNL2572CIR122B, NAU2291B
c5	21	2	7 RKN, 3 VW, 1 BRR	RKN	10–35 cM	CIR067, CIR224a, CIR102, CIR280b, CIR062a
c5			5 VW	VW	75–100 cM	CIR373, BNL3995, JESPR065b
c6	8	1	5 FW, 1 VW	FW	5–25 Cm	CIR267a, BNL3359b, CIR298, CIR203, BNL2569
c7	25	3	10 RKN, 2 VW, 1 FW	RKN	0–25 Cm	pAR057b, pAR188b, CG05a
c7			4 RKN	RKN	40–55 cM	CG23a, CIR355, NAU2432b
c7			5 VW	VW	65–85 cM	NAU2186a, R2, BNL1597, CIR412
c8	14	2	2 FW, 2 VW		0–20 cM	CIR244, G1114, JESPR232a, pAR792a
C8			7 VW	VW	35–60 Cm	CIR376a, JESPR066, CIR237, CIR254a, BNL2961a
c9	17	1	3 VW, 2 RKN		35–55 cM	MUSB1040b, BNL3799, JESPR230b
c10	0	0				
c11	23	2	17 RKN, 1 VW, 1 FW	RKN	0–25 cM	CIR069b, pAR044b, pAR648b
c11			4 RKN, 2 FW, 1 RN^a^	RKN	20–45 cM	pAR044b, pAR648b, CIR196, CIR003, BNL1066, BNL0836
c12	3	0				
c13	6	0				
c14	14	1	9 RKN, 1FW	RKN	0–20 cM	CIR246, pAR723b, G1012
c15	8	1	4 RKN, 1 VW	RKN	40–60 cM	pAR015b, BNL2646, JESPR205
c16	21	3	8 VW	VW	0–25 cM	A1826, pAR544
c16			6 VW, 1 FW	VW	30–55 cM	HAU0966a, BNL1604a, BNL1122b
c16			6 VW	VW	57–75 cM	JESPR228a, HAU2432a, BNL2986
c17	7	1	4 FW, 2 VW	FW	0–10 cM	BNL3408a, CIR347, BNL2443a
c18	2	0				
c19	21	2	5 VW, 5 FW	VW, FW	0–25 cM	CIR415a, CIR224b, CIR242, CIR165a
c19			4 RKN, 2 VW	RKN	20–50 cM	CIR165a, BNL3452, CIR086a, CIR176
c20	7	1	3 VW, 2 RKN		0–20 cM	A1214a, CMS21b, CIR187
c21	22	2	5 VW, 2 RKN, 2 FW	VW	0–25 cM	LTCOL, CG22, CIR112, CIR069a, CIR316, JESPR066a
c21			4 VW		85–105 cM	BNL3997, CIR061c, HAU4855, BNL1053a, BNL1681b
c22	9	1	2 VW, 2 FW, 1 RKN		0–25 cM	NAU2235b, BNL3873, CIR218b, CIR122a, NAU2291a
c23	36	2	22 VW, 1 FW, 2 RKN	VW	0–25 cM	CIR198, CIR286, BNL086b, CIR019, BNL1161a, BNL3383
c23			4 VW	VW	35–45 cM	pAR517, CAC263d
c24	4	1	3 VW, 1 FW		0–25 cM	CIR026, CIR388, JESPR157a, HAU2407b, BNL3860
c25	2	0				
c26	7	1	4 VW	VW	0–20 cM	JESPR300b, NAU3896, CIR272b, BNL1045a

*BRR* black root rot

^a^a major resistance gene *Ren* ^*lon*^ transferred to Upland from *G. longicalyx*

Comparing the distribution of QTL clusters between homeologous chromosomes (Table 3), c1, c2, c3, c10, and c12 did not have any clusters identified, while their counterparts (c15, c14, c17, c20 and c26, respectively) each had one cluster. However, c6 had one cluster while its homeologous chromosome c25 carried no cluster, nor the homeologous pair c13 and c18 carried any clusters. Homeologous chromosome pair c4 and c22 each carried one cluster in similar regions with QTLs for VW and RKN resistance identified, while other three pairs (c5 vs. c19, c7 vs. c16, and c11 vs. c21) each carried 2–3 clusters. For c5 and c19, 3–5 VW QTLs were identified in the similar region (0–30 cM), while the second cluster differed in map positions and types of QTLs between the two chromosomes. For c7 and c16, the first cluster had 2 VW QTLs; the second cluster differed in QTL types, while the third cluster had the same QTL type but differed in positions. For c11 and c21, the first cluster in a similar position only had 2 QTLs in common for the same traits, while the second cluster differed in QTL type and position. Interestingly, for homeologous pair c9 and c23, both had 3 VW QTLs in a similar region (30–50 cM). However, c23 carried 2 clusters (with 25 and 4 QTLs, respectively), while c9 carried one cluster with only 5 QTLs. For another homeologous pair c8 (with 2 clusters) and c24 (with one), both had QTLs for VW and FW resistance in the same region (0–20 cM).

### Meta-analysis of Verticillium wilt (VW) and Fusarium wilt (FW) resistance QTLs

Of a total of 193 QTLs for VW resistance, 83 and 110 were distributed on the A- and D- subgenomes, respectively (Table 2, Fig. 1). But the difference in QTLs between the two subgenomes was not statistically significant (*χ*^2^ = 3.78; χ^2^_0.05_ = 3.84 at df = 1). Except for chromosomes c10 and c18, all chromosomes carried VW resistance QTLs. Chromosome c23 carried the most QTLs (33), followed by c16 with 20 and c21 with 16. Chromosomes c5, c7, c8, c9 and c19 each carried 11–12 QTLs, while c4 and c26 carried 7–8 QTLs. Due to the concentration of QTLs in regions, c4 (at 0–10 cM), c5 (at 80–100 cM), c7 (at 65–85 cM), c8 (35–55 cM), c19 (at 0–25 cM), and c26 (at 0–20 cM) each carried one hotspot with 4–7 VW resistance QTLs, while c21 and c23 each carried 2 hotspots, and c16 carried 3 hotspots (Table 3). On c16, the three hotspots were distributed at 0–20, 30–40, and 50–65 cM with 8, 6 and 6 QTLs, respectively. On c21, at two distal regions (at 0–25 cM and 85–105 cM) from the centromere, the two hotspots each contained 4–5 QTLs. On c23, the hotspot at 0–25 cM had 22 QTLs concentrated for VW resistance, while the one at 35–45 cM had only 4 VW resistance QTLs. The above 13 hotspots contained 86 individual VW resistance QTLs (45 % of total VW QTLs) and a few QTLs for FW or RKN resistance. Of the 193 VW resistance QTLs identified, 111 (57 %) were located in disease resistance QTL clusters.

All 27 FW resistance QTLs reported on 16 chromosomes were located in resistance QTL clusters, 14 of which were located on three chromosomes c6, c17 and c19 (Table 2, Fig. 1). The relevant regions (0–25 cM) on these three chromosomes (5 QTLs at 5–25 cM on c6, 4 QTLs at 0–10 cM on c17, and 5 QTLs at 0–25 cM on c19) each carried a QTL hotspot for FW resistance (Table 3). It is interesting to note that the same region on c19 also carried a QTL hotspot with 5 QTLs for VW resistance. Therefore, this region may have resistance genes for both VW and FW.

### Meta-analysis of root-knot nematode (RKN) and reniform nematode (RN) resistance QTLs

For RKN resistance, a total of 75 QTLs were identified on 14 chromosomes (Table 2, Fig. 1), 18 of which were located on c11, followed by c7 (13 QTLs), c14 (9 QTLs), c5 (7 QTLs), c19 (6 QTLs), c9 (4 QTLs), and c15 (4 QTLs). Chromosomes c1, c3, c4, c20, c21, c22 and c23 each carried 1–3 RKN resistance QTLs, and the remaining chromosomes did not carry any RKN resistance QTLs. Eight QTL hotspots were identified including two each on c7 and c11 (at 0–25 and 20–45 cM regions), and one each on c5 (at 10–35 cM), c14 (at 0–20 cM), c15 (at 40–60 cM), and c19 (at 20–50 cM). Two major RKN resistance genes or QTLs with major effects from Auburn 623RKN and its derived lines, as reported previously [13, 14], have been confirmed in the hotspot regions of c11 with 17 QTLs and c14 with 9 QTLs. Of the 75 RKN QTLs identified, 69 (92 %) and 59 (79 %) were located within 14 resistance QTL clusters and the 8 RKN resistance QTL hotspots, respectively (Table 3).

Of the six QTLs reported for reniform nematode (RN) resistance, c21 carried five, while c18 carried one. Since the five QTLs on c21 were scattered along the whole chromosome, there was no QTL hotspot identified. However, c11 carried one major dominant resistance gene *Ren*^*lon*^ transferred from *G. longicalyx*, and it was in a close proximity to the two RKN resistance QTL hotspots with 21 QTL identified including one of the two major resistance genes for RKN resistance (Table 3, Fig. 1). Therefore, this c11 region carries important resistance genes for both RKN and RN resistance.

### QTLs for resistance to bacterial blight (BB) and black root rot (BRR)

Due to the RFLP markers used by Wright et al. [28], the BB resistance QTLs could not be placed on the consensus map in this study. However, three reported QTLs resistant to BRR were mapped onto c5, c9 and c13 of the consensus map (Fig. 1). The one on c5 was close to a QTL cluster with 7 RKN and 3 VW QTLs, while the other two QTLs were distant from others.

### Identification of linked markers to the disease resistance QTL clusters and hotspot

The closely linked SSR markers for the 28 disease resistance QTL clusters involving 13 VW resistance hotspots, 3 FW resistance hotspots and 7 RKN resistance hotspots are listed in Table 3. The information should be useful to breeders and geneticists.

### Association between number of resistance QTLs and number of nucleotide-binding site (NBS)-encoding genes

Based on the sequenced genomes in *G. raimondii* (D5 genome) and *G. arboreum* (A2 genome), 391 and 280 NBS-coding genes were identified, respectively [24]. The significantly higher number of NBS genes in *G. raimondii* than in *G. arboreum* (*χ*^2^ = 18.36; χ^2^_0.05_ = 3.84 at df = 1) may explain why the D subgenome carried more disease resistance QTLs than the A subgenome in the tetraploid cotton. The number of NBS genes and the number of QTLs identified on the 13 D-subgenome chromosomes were positively correlated (*r* = 0.399; *P* > 0.05), while the correlation was significant with the number of total disease resistance QTLs on homeologous A-subgenome chromosomes (*r* = 0.645; 0.01 < *P* < 0.05). The sum of QTLs in 13 pairs of homeologous chromosomes was also significantly correlated with the number of NBS-coding genes (*r* = 0.553; 0.01 < *P* < 0.05). The correlation with the number VW QTLs on A- and D- subgenome chromosomes was also positive but insignificant (*r* = 0.387–0.464; *P* > 0.05); however, the correlation with the number of RKN QTLs on the A subgenome chromosomes was significant (*r* = 0.645; 0.01 < *P* < 0.01). Therefore, there is a trend that the more NBS-encoding genes a chromosome carries, the more disease resistance QTLs it has. For example, chromosome 7 (D5) carried the most number of NBS genes (87), and its homeologous pair of Upland cotton (c7 vs. c16) carried a total of 46 QTLs (25 vs. 21). Chromosomes 9 and 11 in D5 carried 23 and 34 NBS genes, respectively, and their homeologous pairs (c9 vs. c23, and c11 vs. c21) carried a total of 53 (17 vs. 36) and 46 (23 vs. 23) QTLs, respectively. Chromosomes 6, 10, 12 and 13 in D5 contained a minimum numbers of NBS genes (1–8), their corresponding tetraploid chromosomes (c6 vs. c25, c10 vs. c20, c12 vs. c26, and c13 vs. c18) also carried the least numbers of QTLs (8 vs. 2, 0 vs. 7, 3 vs. 7, and 6 vs. 2, respectively). However, three chromosomes (1, 2, and 8) in D5 also had high numbers of NBS genes (24, 22, and 32, respectively), but their tetraploid counterparts (c1 vs. c15, c2 vs. c14, and c8 vs. c24, respectively) only carried moderate numbers of QTLs (5 vs. 8, 5 vs. 14, and 14 vs. 4, respectively). Surprisingly, chromosomes 5 in D5 carried a small number of NBS genes (5), but its tetraploid counterparts (c5 vs. c19) carried a high number of QTLs (21 vs. 21).

Furthermore, the number of NBS genes was significantly correlated with the number of QTL clusters on A- and D- subgenome chromosomes and the sum of the homeologous chromosomes (*r* = 0.683, *r* = 0.710, and *r* = 0.754, respectively; r_0.05_ = 0.553, r_0.01_ = 0.684; df = 11). For example, chromosomes 7, 8, 9 and 11 with high numbers of NBS genes each had a total of 3–6 clusters in their tetraploid counterparts. Since NBS genes on these chromosomes with high numbers of NBS genes are clustered, the results indicate that the disease resistance QTL clusters may be in part determined by NBS gene clusters. However, the reverse is true for chromosome 5 with only 5 NBS genes in the D5 genome, while its tetraploid counterparts carried 4 clusters. This result indicates that either these NBS genes may have pleiotropic effects on multiple diseases or other genes on the chromosome are involved in quantitative resistance.

## Discussion

### Difficulties in screening cotton for VW resistance

In this study, a BIL population of 146 lines was tested in 2–4 replications (with 27–30 plants for each genotype in each replication) through 4-years replicated field tests. Disease resistance was determined by an average severity rating from 54–120 individual plants for each genotype, therefore rendering low experimental errors than most previous VW resistance studies. However, only 10 VW resistance QTLs on 8 chromosomes were identified. On two chromosome regions, two QTLs were identified in the same or similar regions. On the 8 chromosomes where the 10 QTLs were mapped, VW resistance QTLs were also mapped by previous studies. In fact, 4 QTLs were mapped onto three chromosomes (c4, c21 and c23) with VW QTL hotspots. Therefore, the results in this study demonstrated a moderate level of consistency in QTL mapping for VW resistance based on the multiple years of replicated studies. The results also demonstrated difficulties in VW resistance studies even if inbred lines are evaluated for VW resistance in multiple replicated field tests with or without inoculations.

As Zhang et al. [4, 10] noted, it is difficult to reliably identify VW and FW resistance QTLs. There are a number of contributing factors to the complication of disease resistance studies. First, Many early segregating populations were used in disease resistance QTL mapping, which did not allow repeated evaluation of the same genotypes from multiple individuals in multiple replications and multiple environments. Experimental errors were understandably higher. Second, VW and FW disease infections are highly sensitive to environmental and developmental factors, and even artificial inoculations could not achieve similar disease infections in the same genotypes [4, 8, 29, 30]. Thirdly, an artificial grading system for disease severity is often used, rendering it very difficult to quantitatively and accurately phenotype cotton responses to the diseases. Fourth, there exist interactions of genotype with environment, strains of a pathogen, and evaluation methods, resulting in different disease responses of the same genotypes to different strains of pathogens under different environmental conditions or using different inoculation methods. Furthermore, most disease resistance QTLs have low contributions to disease resistance, which could not be detected in many environmental conditions where experimental errors for resistance screening are higher. Finally, low genome coverage of molecular markers in many mapping studies does not allow a genome-wide detection of QTLs with a high resolution, resulting in different QTLs identified from different genetic populations evaluated under different environmental and screening conditions. All of these issues call for reliable screening techniques and phenotyping of disease resistance in mapping populations with multiple individuals in each genotype using multiple replicated tests. Of course, genome-wide markers should be developed for better genome coverage, so more QTLs with high accumulated PVE will be detected.

### Genetic basis of VW resistance

The complexity in mapping QTLs for VW resistance in cotton is further illustrated from the meta-analysis of VW resistance QTLs reported previously. Out of 193 QTLs reported, except for c10 and c18 where no QTLs for VW resistance were reported, all other chromosomes had QTLs mapped, although 14 chromosomes carried only 1–6 VW QTLs. The identification of 13 VW resistance QTL hotspots on 9 chromosomes further demonstrated the complexity in studying the genetic basis of VW resistance in cotton, because selection of QTLs for VW resistance breeding and further genetic and genomic studies will be difficult. However, more attention should be paid to several VW QTL populated hotspots, such as the hotspot with 7 VW QTLs on c8 at 35–60 cM and three hotspots with 22 VW QTLs on c16 at 0–75 cM. But, the most notable is the hotspot with 22 VW QTLs on c23 at 0–25 cM, because this region may also confer resistance to FW and RKN since 1–2 resistance QTLs to FW and RKN were identified.

Several other regions also deserve more attention. The VW QTL hotspot on c19 is interesting, because a FW QTL hotspot was within the same region, and a RKN QTL hotspot was also in the proximity. Therefore, this region may share genes responsive to VW, FW and RKN.

A region on c11 is also very important in conferring resistance to multiple diseases. In a broad region (0–45 cM), 21 RKN QTLs, 3 FW QTLs, 1 VW QTL, and 1 major RN resistance gene (*Ren*^*lon*^) were mapped. Another region (0–20 cM) with the second most frequent RKN QTLs was on c14. In this region, a major gene or QTL was identified for RKN resistance [13, 14]. However, this region appeared to confer resistance to only RKN, because there were very few QTLs identified for resistance to other diseases.

Chromosome c21 also deserves more consideration, because it carried two QTL hotspots for VW resistance, 2 QTL for RKN resistance and also 5 QTLs for reniform nematode (RN) resistance. However, the RN resistance QTLs were scattered along the chromosome and not contained in any of the hotspots. Except for the hotspot in the same region on c19 for VW and FW resistance, other hotspots including 8 hotspots for RKN resistance did not overlap with any of the resistance QTL hotspots for VW and FW. In fact, the numbers of QTLs for VW, FW and RKN on different chromosomes were not correlated (*r* = −0.039 to 0.114; r_0.05_ = 0.388 at df = 24). Therefore, it is reasonable to speculate that the resistance mechanisms for the three diseases are likely different. However, the linked markers for 13 VW hotspots, 3 FW hotspots and 8 RKN hotspots should be highly useful in choosing chromosome regions with consistent QTLs for marker-assisted selection and high resolution mapping of resistance QTLs and genes.

### NBS-coding genes and disease resistance QTLs

In rice and other plant species, many disease resistance genes were cloned and isolated [22, 23]. It is known that most of the disease resistance genes belong to a super gene family encoding nucleotide-binding site (NBS) domains. Through resistance gene analog RGA-based marker analysis, many RGAs in cotton were mapped [31-33]. Except that a RGA marker was found to be linked to a RKN resistance gene in a study [32], no other studies have associated NBS genes with disease resistance in cotton. Based on the current study, the number of disease resistance QTLs and QTL clusters including hotspots identified on chromosomes seemed to be positively correlated with the number of NBS genes. However, on several other chromosomes with very few NBS genes, substantially higher numbers of resistance QTLs were identified. It is likely that in some QTL cluster and hotspot regions, multiple NBS genes are located, and different NBS genes may confer resistance to different diseases. Since NBS-coding R genes are normally major Mendelian resistance genes [22, 23], the positive correlation of QTL clusters and hotspots identified in the current meta-analysis with NBS genes indicates that these QTL regions maybe contain major resistance R genes. In fact, major disease resistance genes or QTLs with major effects have been identified for VW, FW, RKN, RN, and BB [4, 10, 12–14, 17, 19–21, 34]. For example, two major resistance genes were identified for RKN [12–14]; and more than 12 major genes resistant to various races of BB including one on chromosome 5 have been reported [34]. Others major resistance genes include one resistant to southwestern cotton rust (*Puccinia ccabata* Arth. and Holw.) and two resistant to cotton leaf crumple virus [34]. Recent studies have shown that some major disease resistance R genes in other plants are co-localized with resistance QTLs, suggesting weak or defeated effects of R genes or their tight linkage with other genes responsible for quantitative resistance loci [35]. There are other genes with different functions identified recently that may be responsible for quantitative disease resistance [35, 36]. Further studies are needed to discern the relationship between disease resistance QTLs and NBS genes in cotton. Because a 25 cM region may contain 700–800 genes based on the sequenced diploid cotton genomes, identification of candidate genes for the resistance QTL clusters is currently impractical in this study. In the future, positional candidate gene approaches in relating NBS genes to resistance QTLs will be possible once the tetraploid cotton genome is sequenced and the QTL clusters are narrowed to 5–10 cM regions through a high resolution mapping strategy using large genetic populations.

## Materials and methods

### Materials

An interspecific backcross inbred line (BIL) population comprising of 146 lines was used in this study. The BILs were developed from a cross between Upland cotton (*G. hirsutum*) SG 747 and *G. barbadense* Giza 75 through two generations of backcrossing using SG 747 as the recurrent parent followed by four generations of self-pollination. During the BIL development, each BC_1_F_1_ plant was used as male parent to backcross with SG 747 to derive BC_2_F_1_. In each BC_1_F_1_-derived BC_2_F_5_ progeny, one single representative plant was selected for seed increase and used as the seed source for subsequent field tests. The 146 BILs and the two parents were planted in the experimental farm of China Cotton Research Institute, Chinese Academy of Agricultural Sciences, Anyang, Henan province in 2006, 2007 and 2008. The fields used to evaluate VW resistance were grown with cotton yearly and heavily infected with race 3 of *V. dahliae* Kleb. To further evaluate VW resistance, an artificially inoculated field nursery with the VW strain from Anyang was used in 2009. The 148 entries were arranged in a randomized complete block design with two (2007), three (2008 and 2009) and four replications (2006). Seeds were sown in single row plots in April and crop managements followed local recommendations. The plot length was 8.3 m with a row-spacing of 0.8 m and seedlings were thinned to 27–30 plants per plot.

### VW resistance screening

All the individual plants in each plot were evaluated for VW resistance based on a system established as a national standard for screening cotton for VW resistance in China [4], as the following,

0. No symptom (healthy)
1. <25 % chlorotic/necrotic leaves
2. 25–50 % chlorotic/necrotic leaves
3. 50–75 % chlorotic/necrotic leaves
4. >75 % chlorotic/necrotic leaves
5. Complete defoliation or plant death

The number of infected plants was divided by the total number of plants screened to calculate disease incidence (VW %), and average disease severity rating-VWR, i.e., the sum of (rating × number of plants) was divided by the total number of plants [4, 7]. The average disease severity rating on the 0–5 scale was converted to the disease index (%) on a 0–100 % scale as the ratio between the average severity rating and the highest rating (i.e., 5).

### DNA extraction, maker analysis, and map construction

The genomic DNAs were extracted from young leaves of the 146 individual BIL lines and the two parents using a quick method [37]. Simple sequence repeat markers (SSRs) were used to construct a genetic map for the BIL population using JoinMap 3.0 [38] and the linkage map was published elsewhere [27].

### QTL mapping

For QTL mapping, the IciMapping software (v3.2; http://www.isbreeding.net/), an integrated software for building linkage maps and mapping QTLs which can handle various mapping populations including BILs in this study, was used [39]. See Yu et al. [27] for details. The QTL nomenclature followed McCouch et al. [40] in that a QTL designation begins with “q”, followed by an abbreviation of the trait name, year, chromosome name, and finally a serial number.

### Meta-analysis of QTLs

As of the end of January 2015, results of disease resistance QTL mapping from all publicly accessible journals were obtained, in addition to a few papers published in Chinese in China. This study used 32 published papers regarding mapping of resistance to VW, FW, RKN, RN, bacterial blight (BB) and black root rot (BRR) with 306 QTLs reported (see Table 4 for details). For VW resistance QTL mapping, 12 studies [41–50] were included. For FW QTL mapping, 6 studies [51–56] were included. For RKN QTL mapping, 7 studies [13, 14, 57–60] were included. For RN QTL mapping, 5 studies were included [17, 19–21]. To be more inclusive, results from two studies on BB and BRR resistance QTL mapping were also used [28, 61].

**Table 4:** Mapping of resistance to Verticillium wilt (VW), Fusarium wilt (FW), root-knot nematodes (RKN) and reniform nematodes (RN) that were identified in this study and reported in other studies

Author	Journal	Year	No. QTL	Population	Disease
Wright RJ et al.	Genetics	1998	2	F2	BB
Niu C et al.	Theor Appl Genet	2008	3	F2/F2:3	BRR
Wang & Roberts	Phytopathology	2006	1	F2:3	FW
Wang PZ et al.	Theor Appl Genet	2009	6	F2:3	FW
Lopez-Lavalle et al.	Mol Breed	2012	9	F3/F4	FW
Mei H et al.	Euphytica	2014	7	4WC	FW
Ulloa M et al.	Mol Genet Genomics	2011	6	RIL	FW
Ulloa M et al.	Theor Appl Genet	2013	4	F2 & RIL	FW
Gutierrez OA et al.	Theor Appl Genet	2010	12	RIL	RKN
He et al.	Theor Appl Genet	2014	1	F2	RKN
Shen X et al.	Theor Appl Genet	2010	1	F2	RKN
Shen X et al.	Theor Appl Genet	2006	13	F2	RKN
Wang C et al.	Theor Appl Genet	2006	1	RIL	RKN
Wang C et al.	PLoS ONE	2012	45	RIL	RKN
Ulloa M et al.	Plant Breed	2009	2	BC1P1/P2	RKN
Buyyarapu R et al.	ICGI 2014 Conf	2014	1	F2	RN
Dighe ND et al.	Crop Sci	2009	1	BCF1/BCS1	RN
Gutierrez OA et al.	Theor Appl Genet	2011	3	BCP1/2	RN
Romano GB et al.	Theor Appl Genet	2009	1	Trispecific	RN
Bolek et al.	Plant Sci	2005	33	F2	VW
Fang H et al.	Mol Breed	2014	19	RIL	VW
Fang H et al.	Euphytica	2013	3	BIL	VW
Ge HY et al.	Cotton Sci	2008	1	F2:3	VW
Jiang F et al.	Sci in China Ser C: Life Sci	2009	41	F2:3	VW
Wang FR et al.	Cotton Sci	2007	4	F2:3	VW
Zhao Y et al.	PLoS ONE	2014	14	AM	VW
Wang P et al.	The Crop Journal	2014	23	CSILs	VW
Wang HM et al.	J Integr Plant Biol	2008	4	F2:3	VW
Zhang X et al.	PLoS ONE	2014	5	F2:3	VW
Yang C et al.	Plant Sci	2008	18	BC1S2	VW
Ning ZY et al.	Crop Sci	2013	12	RIL	VW
Zhang JF et al.	This study	2015	10	BIL	VW

*BB* bacterial blight, *BRR* black root rot, *RIL* recombinant inbred line, *BIL* backcross inbred line, *CSIL* chromosome segment introgression line, *AM* association mapping panel

A meta-analysis of QTLs for VW resistance was performed using Biomercator V3 software ([62] (http://moulon.inra.fr/index.php/fr/equipestransversales/atelier-de-bioinformatique/projects/ projets/135). Briefly, using Biomercator V3, the map file and QTL file from each study were loaded into the software in the tab delimited format, and were then mapped to the consensus [“Guazuncho2” (*G. hirsutum*) × “VH8-4602” (*G. barbadense*)] map [63] obtained from the Cotton Marker Database [64]. Since the map file contains distances between markers on each chromosome, each population’s QTLs were mapped to the consensus map separately. A detailed description in the meta-analysis of resistance QTLs can be found in Said et al. [65, 66].

To reduce errors in declaring a QTL, four or more QTLs (with a false positive rate of 6.25 % or below) in an interval of 25 cM were considered a consistent QTL region. If there was more than one trait involved in the QTLs, the region is called a QTL cluster. Otherwise, it is called a QTL hotspot for the region involving only one single trait.

### Correlation analysis between number of QTLs and nucleotide-binding site (NBS)-encoding genes distributed on chromosomes

Based on the recent completion of genome sequencing of *G. raimondii* and *G. arboreum* [24–26], NBS-encoding genes were identified. For example, chromosomes 1 through 13 of the *G. raimondii* genome carried 24, 22, 11, 8, 5, 5, 87, 32, 23, 5, 34, 1, and 8, respectively [67]. A simple correlation analysis was performed between the number of the NBS genes and the number of total QTLs and QTLs resistant to individual diseases. Coefficients of correlation were tested for significance at the degree of freedom of 24 (genomewide with 26 chromosomes) or 11 (on the subgenome level with 13 chromosomes).

## Conclusions

In this study, linkage mapping of Verticillium wilt (VW) resistance and meta-analysis of QTLs were used to map QTL clusters and hotspots for resistance to VW, Fusarium wilt, root-knot nematodes and reniform nematodes in cotton. In a four-year replicated test of a backcross inbred line population for VW resistance, 10 resistance QTLs were mapped based on a 2895 cM linkage map with 392 SSR markers, which has added useful information to the understanding of the genetic basis of VW resistance in cotton. Twenty-eight disease resistance QTL clusters and 24 hotspots identified from a total of 306 reported QTLs in 32 papers and linked SSR markers provide important information for marker-assisted selection and high resolution mapping of resistance QTLs and genes. The non-overlapping of most resistance QTL hotspots for different diseases indicates that their resistances are controlled by different genes.

## Additional file

Additional file 1:
**Mapping of quantitative trait loci for Verticillium wilt resistance in a backcross inbred line population of (SG 747 × Giza 75) × SG 747 BC**_**2**_ **F**_**4**_ **.**

## Abbreviations

BB: Bacterial blight
BIL: Backcross inbred line
BRR: Black root rot
FW: Fusarium wilt
NBS: Nucleotide binding site
QTL: Quantitative trait locus (QTL)
RKN: Root-knot nematodes
RN: Reniform nematodes
SNP: Single nucleotide polymorphism
SSR: Simple sequence repeat
VW: Verticillium wilt
