# Supplementary material for: Genetic analysis of Verticillium wilt resistance in a backcross inbred line population and a meta-analysis of quantitative trait loci for disease resistance in cotton
Source: BMC Genomics. 2015 Aug 5;16(1):577. doi: 10.1186/s12864-015-1682-2 (PMC4524102; doi:10.1186/s12864-015-1682-2)
Supplement: Additional file 1: — Mapping of quantitative trait loci for Verticillium wilt resistance in a backcross inbred line population of (SG 747 × Giza 75) × SG 747 BC 2 F 4 . [file 12864_2015_1682_MOESM1_ESM.docx]

**Additional file 1.** Mapping of quantitative trait loci for Verticillium wilt resistance in a backcross inbred line population of (SG 747 × Giza 75) × SG 747 BC_2_F_4_.
